# The impact of glucose and metabolic disturbances on white matter hyperintensity volume in apparently healthy adults

**DOI:** 10.3389/fendo.2026.1789777

**Published:** 2026-03-18

**Authors:** Aleksandra Szum-Jakubowska, Marlena Dubatówka, Małgorzata Chlabicz, Jacek Jamiołkowski, Marcin Hładuński, Alexander Teumer, Katharina Wittfeld, Mikołaj A. Pawlak, Irina Kowalska, Karol A. Kamiński

**Affiliations:** 1 Department of Population Medicine and Lifestyle Diseases Prevention, Medical University of Bialystok, Bialystok, Poland; 2 Population Research Center, Medical University of Bialystok, Bialystok, Poland; 3 Department of Invasive Cardiology, Medical University of Bialystok, Bialystok, Poland; 4 Independent Laboratory of Molecular Imaging, Medical University of Bialystok, Bialystok, Poland; 5 Department of Psychiatry and Psychotherapy, University Medicine Greifswald, Greifswald, Germany; 6 DZHK (German Centre for Cardiovascular Research), Partner Site Greifswald, Greifswald, Germany; 7 Department of Neurology, Poznan University of Medical Sciences, Poznan, Poland; 8 Department of Clinical Genetics, Erasmus Medical Center, Rotterdam, Netherlands; 9 Department of Internal Medicine and Metabolic Diseases, Medical University of Bialystok, Bialystok, Poland; 10 Department of Cardiology, Medical University of Bialystok, Bialystok, Poland

**Keywords:** brain aging, diabetes mellitus, metabolic syndrome, neurodegeneration, prediabetes, white matter hyperintensities

## Abstract

**Background:**

White matter hyperintensities (WMHs), commonly seen in brain magnetic resonance imaging (MRI), are linked to cognitive decline and may be influenced by cardiovascular risk factors. This study explores the relationship between diabetes, prediabetes, metabolic syndrome, and the presence of WMHs in an apparently healthy population.

**Methods:**

The study group includes 735 adult participants without neurological or severe cardiovascular diseases. During the visit, participants took part in detailed clinical examination (medical history, biochemical analysis, carotid arteries ultrasound, and brain MRI). WMHs were quantified by the SAMSEG tool implemented in Freesurfer software.

**Results:**

Participants’ median age was 45 (range, 36–58) years, 341 (46.39%) were men, 58 (7.9%) had diagnosed diabetes, 345 (46.94%) had diagnosed prediabetes, and 91 (12.38%) individuals fulfilled two definitions of prediabetes—simultaneously impaired glucose tolerance (IGT) and impaired fasting glucose (IFG). Univariate analysis presented a positive association between plasma glucose concentrations, glycated hemoglobin, diabetes mellitus, prediabetes, and metabolic syndrome and WMHs (*p* < 0.05). Multivariate analysis (*R*^2adj.^ = 0.33) presented an association between glucose metabolism disorders (diabetes mellitus or fulfilling two definitions of prediabetes, β = 2.77, *p* = 0.006) and WMHs.

**Conclusion:**

Patients with glucose metabolism disorders, not only those with diabetes but also those fulfilling definitions of prediabetes IGT and IFG simultaneously, have significantly larger volumes of WMHs. Patients with diabetes or prediabetes may benefit from the comprehensive management of cardiovascular risk factors, not only to limit the risk of cardiovascular disease but also to potentially reduce the risk of cognitive impairment.

## 1 Background

Cognitive decline refers to the progressive worsening of memory, thinking, and reasoning skills, commonly seen with advancing age. While aging itself increases the risk, this process is further influenced by genetics, lifestyle habits, and health problems (1). A key role is played by the central nervous system, where complex neural circuits regulate cognitive performance. Damage to these circuits—caused by conditions like Alzheimer’s disease or vascular issues such as stroke or atherosclerosis—can significantly speed up the decline in cognitive abilities (2). White matter hyperintensities (WMHs), which are non-specific white matter (WM) lesions appearing as bright signals on T2-weighted or FLAIR MRI, are associated with aging, cardiovascular risk (CVR) factors (e.g., hypertension, dyslipidemia, and diabetes), and increased risk of cognitive decline, dementia, and stroke (3–5).

Diabetes mellitus is a chronic metabolic disease characterized by hyperglycemia. Prediabetes, representing an early stage in the development of diabetes, can be diagnosed as impaired fasting glucose (IFG) or impaired glucose tolerance (IGT) with diagnostic criteria based on American Diabetes Association (ADA) guidelines (outlined in **Table 1**) (6). Obesity, particularly visceral obesity, is a key risk factor for type 2 diabetes mellitus, as it contributes to insulin resistance and chronic inflammation (6, 7).

**Table 1:** Diagnosis criteria for diabetes mellitus and prediabetes status based on ADA criteria (6).

Condition	Diagnosis criteria
Diabetes	Fasting glucose ≥ 126 mg/dL in two measurements in two different days * or 2 h post glucose load ≥ 200 mg/dL or glycated hemoglobin ≥ 6.5%
Isolated impaired fasting glucose (isolated IFG) **	No previous diabetes diagnosis and fasting glucose between 100 and 125 mg/dL and 2 h post glucose load < 140 mg/dL
Isolated impaired glucose tolerance (isolated IGT) **	No previous diabetes diagnosis and fasting glucose < 100 mg/dL and 2 h post glucose load between 140 and 199 mg/dL
Impaired fasting glucose with impaired glucose tolerance (IGT + IFG) ***	No previous diabetes diagnosis and fasting glucose between 100 and 125 mg/dL and 2 h post glucose load between 140 and 199 mg/dL
Prediabetes	No previous diabetes diagnosis and fasting glucose between 100 and 125 mg/dL or 2 h post glucose load between 140 and 199 mg/dL or glycated hemoglobin between 5.7% and 6.4%

*In our study, diabetes was not diagnosed based on fasting glucose due to availability of a single measurement only.

**Prediabetes I group: isolated IFG + isolated IGT.

***Prediabetes II group.

Metabolic syndrome is defined as a condition characterized by the coexistence of obesity, particularly abdominal obesity with its complications and hypertension, higher triglyceride level, lower high-density lipoprotein, and diabetes or prediabetes status. In 2022, Polish researchers proposed the new metabolic syndrome definition, which is a combination of obesity and CVR factors (8). The basic criterion is abdominal obesity as a waist circumstance ≥ 102 cm for men or ≥ 88 cm for women or body mass index ≥ 30 kg/m^2^. In addition, the individuals must meet two of the following criteria:

- Prediabetes or diabetes status based on ADA guidelines (6) or glucose-lowering treatment,
- Elevated non-high-density lipoprotein cholesterol level (≥130 mg/dL) or lipid-lowering treatment, and
- High normal blood pressure or hypertension (≥130/85 mmHg in the doctor’s office or ≥130/80 mmHg at home) or antihypertensive treatment.

Our previous study based on data from the Bialystok PLUS cohort presented that WMHs are significantly associated with higher CVR categories, carotid plaques, central systolic blood pressure, and glycated hemoglobin (9). While obesity correlates with WMH in univariable analysis, this link disappears in multivariable models, likely due to its inclusion in CVR assessment. Even after excluding individuals with diabetes or hypertension, high CVR and increased intima-media thickness remain associated with WMH, highlighting the positive correlation between asymptomatic carotid atherosclerosis and WMH.

Our objective was to evaluate the association between glycemic status, metabolic syndrome, and the burden of WMH, while accounting for the influence of established CVR factors in a cohort of potentially healthy individuals from the adult population enrolled in the Bialystok PLUS study. We hypothesized that body fat contributes to the development of glycemic abnormalities, which, in turn, may promote the formation of WMH.

## 2 Methods

### 2.1 Population

**Figure 1:**
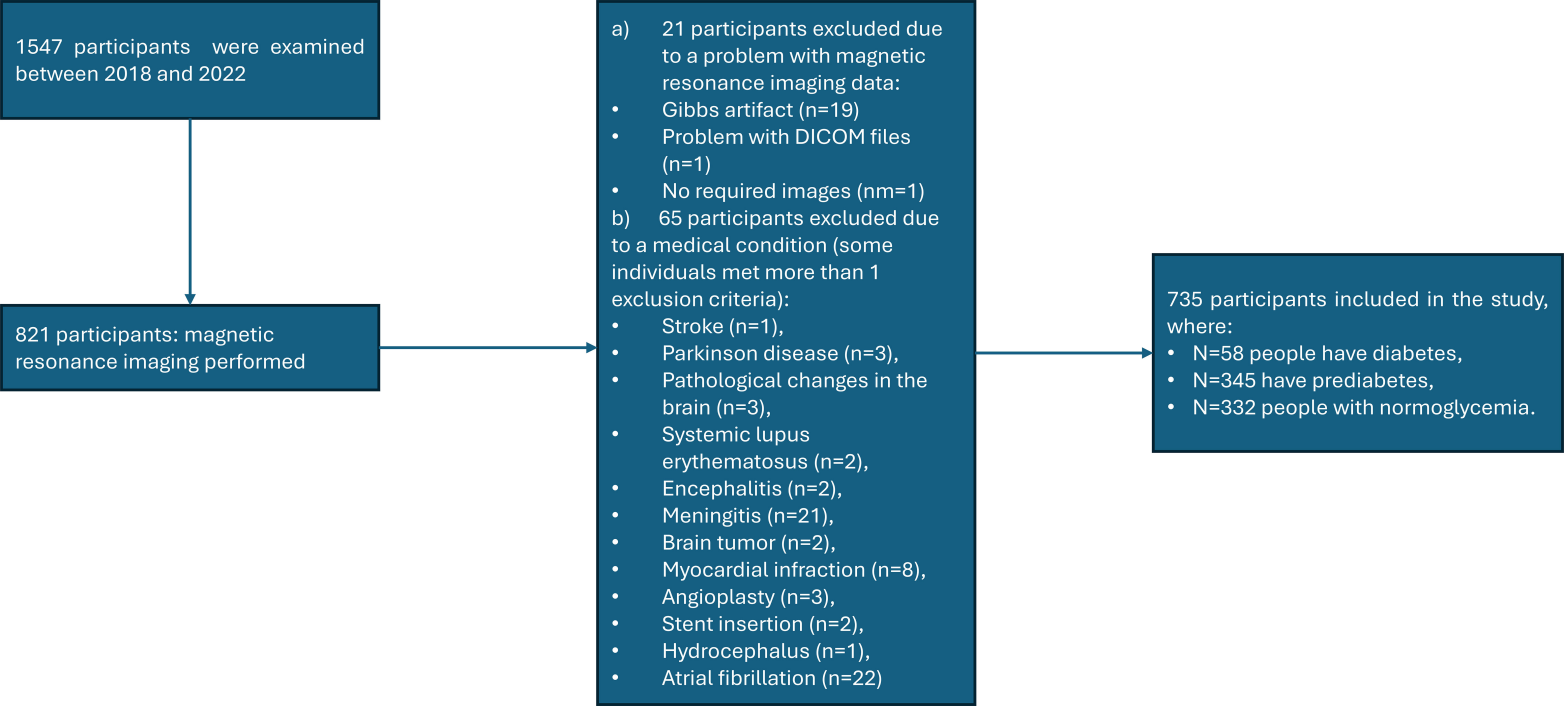
Study group selection from a population cohort study.

This research was part of the Bialystok PLUS cohort study (10), which was implemented at the Population Research Centre, Medical University of Bialystok. The participants were randomly selected from Bialystok residents aged 20–79, based on age and gender to represent the demographic structure of the general population of a middle-sized city—Bialystok. Those who did not have typical contraindications to MRI were offered MRI scanning. Finally, the study group contained 735 apparently healthy participants with high-quality brain MR image and without neurological disorders such as pathological changes in brain, hydrocephalus, previous stroke, Alzheimer’s disease, Parkinson’s disease, systemic lupus erythematosus, encephalitis, meningitis, and brain tumor; without severe cardiac disease such as heart failure, myocardial infarction, and atrial fibrillation; and without cardiac interventions such as angioplasty and stent implantation as previously described (9) and presented in **Figure 1**. In this study, the term “apparently healthy” was used to describe community-dwelling adults without overt neurological disease or clinically manifest severe cardiovascular disease; however, the presence of common cardiometabolic conditions (e.g., hypertension, diabetes, or dyslipidemia) was not an exclusion criterion.

### 2.2 Data collection

Data were collected by qualified medical staff. Participants’ health status and demographic details were obtained using standardized questionnaires (11).

Blood samples were collected in the morning after a minimum of 8 h of fasting. People who were not previously diagnosed with diabetes participated in the oral glucose tolerance test (OGTT). The following parameters were measured: fasting glucose, glucose 120 min after OGTT, glycated hemoglobin, fasting C-peptide, total cholesterol level, and triglycerides. Collected samples were analyzed with the Cobas device (Roche Diagnostics, Meylan, France). Hypercholesterolemia was defined as a history of hypercholesterolemia or cholesterol level ≥ 200 mg/dL. Diabetes mellitus was defined as a history of diabetes mellitus or glucose 120 min after OGTT ≥ 200 mg/dL or glycated hemoglobin level ≥ 6.5%. In our study, diabetes was not diagnosed based on fasting glucose due to the availability of a single measurement only. Individuals who did not suffer from diabetes mellitus were divided into “normoglycemic” and two prediabetes groups (“prediabetes I”: isolated IGT or isolated IFG, or “prediabetes II”: fulfilling criteria of both IGT and IFG) based on ADA (6) guidelines. Owing to the lack of clear criteria for the diagnosis of IGT or IGF based solely on the value of glycated hemoglobin, only criteria related to the need for treatment in fasting blood and after glucose loading are used. Since this study evaluates the population of Bialystok—a Polish population—the criteria for assessing metabolic syndrome were based on the guidelines proposed by Polish experts in 2022 (8).

Systolic and diastolic blood pressure were measured by using the Omron M6 AC Comfort device (Omron Healthcare CO., Ltd., Kyoto, Japan). Hypertension was classified as a history of hypertension or systolic blood pressure ≥ 140 mmHg or diastolic blood pressure ≥ 90 mmHg.

Carotid ultrasound examination was performed by using a Vivid™ E9 ultrasound scanner (GE Healthcare, Chicago, IL, USA). Examination was performed on both sides and assessed the presence of any atherosclerotic plaques in the internal and external carotid artery, common carotid artery, carotid bifurcation, and the presence of carotid plaques in the carotid based on Manheim’s consensus (12). Intima-media complex thickness for the left and right side was measured. Mean intima-media thickness was calculated from left and right measurements.

Anthropometric measurements were performed by the medical staff. Body weight and body composition were measured using an Inbody 770 device (InBody Co., Ltd., Seoul, Korea), and height was measured by a SECA 264 Height Gauge (SECA, Hamburg, Germany). By using SECA 201 tapes (SECA, Hamburg, Germany), we assessed waist and hip circumference. Body mass index was calculated by using the formula $\text{BMI }[\frac{\text{kg}}{{\text{m}}^{2}}]=\;\frac{\text{weight }[\text{kg}]}{{\left(\text{height}\right)}^{2}{[\text{m}}^{2}]}$ and waist-to-hip ratio was determined by using the formula $\text{WHR}=\;\frac{\text{waist circumference }[\text{cm}]}{\text{hip circumference }[\text{cm}]}$. Obesity was defined as BMI ≥ 30.00, and increased WHR index was defined as WHR ≥ 0.85 for women and WHR ≥ 0.90 for men and increased waist circumference > 88 cm for women and > 102 cm for men. The study group participated in dual-energy x-ray absorptiometry (DEXA), which was used to measure bone mineral density and body composition, with body mass divided into 3 compartments: bone, fat mass, and lean mass. DEXA was performed using Lunar iDXA (GE Healthcare, Chicago, Illinois, USA).

Whole-body magnetic resonance imaging (MRI) was performed at the Independent Laboratory of Molecular Imaging at Medical University of Bialystok within a month from the initial visit. It utilized 3-Tesla Siemens Biograph™ mMRI (Siemens, Erlangen, Germany), and five brain imaging protocols were performed. For the purpose of this study, we analyzed the 3D T1-weighted MPRAGE (magnetization prepared rapid gradient echo) structural brain images with TR = 2,300 ms, TE = 2.3 ms, layer thickness = 1 mm, matrix size 176 × 256 × 256, and voxel size 1 × 0.977 × 0.977 mm^3^, and the 2D T2-weighted FLAIR (fluid attenuated inversion recovery) images, with the following parameters: TR = 9,000 ms, TE = 94 ms, layer thickness = 4 mm, matrix size 512 × 416 × 27, and voxel size 0.47 × 0.47 × 5.2 mm^3^.

We performed visual quality control assessment. Next, the morphometric brain structure was analyzed using the Freesurfer version 7.2.0 tool (available at https://surfer.nmr.mgh.harvard.edu/) using a *recon-all* stream on T1-weighted brain images (13). We obtained the WM volume. To estimate the distribution of WMH volume, we performed an analysis using the SAMSEG tool implemented in the Freesurfer tool (14). The value obtained—WMH volume—is characterized by a right-skewed distribution. Next, we calculated the ratio of WMH volume to WM volume and then performed a transformation ln(*x*/(1 − *x*)), where *x* is the percentage of WMH to express the burden of WMH on a logit scale (hereinafter referred to as WMH burden). Because of the non-normal distribution and the presence of extreme values, we additionally applied a percentile rank transformation to rescale the WMH burden variable—and we gained the percentiles of the WMH burden variable. This approach preserves the rank information while mitigating the influence of outliers and making the variable more suitable for statistical modeling by approximating a uniform distribution. The transformation also facilitates comparability between individuals and improves the interpretability of the WMH burden in subsequent analyses.

### 2.3 Statistical analysis

The normal distribution for continuous variables was checked by the Shapiro–Wilk test. Descriptive statistics for quantitative variables were presented as median and quartile range, and as counts and frequencies for qualitative variables. We divided the study group into three groups based on the quartile of WMH burden (the first group was below the median value, the second group was on the third quartile, and third group was on fourth quartile). Comparisons of continuous variables between subgroups were conducted using the Kruskal–Wallis test because of non-normal distribution for continuous variables. Comparisons of categorical variables between subgroups were conducted using the Pearson’s chi-square test. For *post-hoc* tests, we used the Dunn’s test with Bonferroni’s corrections. For significant factors from the univariate analysis, we built linear regression models adjusted for age and sex to examine their association with WMH percentile. The results were consistent after accounting for heteroskedasticity. To address concerns regarding interpretability and robustness, we performed a sensitivity analysis using log-transformed WMH volume (log[WMH volume+1]). The results of the sensitivity analysis are largely consistent with the main analysis: the direction of effects is maintained for most predictors, although some effects are attenuated or become nonsignificant. This supports the fact that our main findings are robust, while percentiles provide a more reliable basis for linear modeling given the skewed distribution. Next, we performed the exploratory analyses by using the appropriate visualization tools and the Kruskal–Wallis, Mann–Whitney *U*, or chi-square test. Before using machine learning methods, continuous variables had been normalized (so that mean = 0 and standard deviation = 1).

We initially constructed multiple linear regression models to examine the associations between glucose disturbance groups or metabolic syndrome and WMH percentiles, adjusting for key covariates such as age, sex, and hypertension. The first three models assessed the association between exposure (diabetes, diabetes or “prediabetes II”, diabetes or “prediabetes I” or “prediabetes II”) and WMH percentiles. The fourth assessed the association of exposure—the presence of metabolic syndrome—on WMH percentiles. These models allowed an initial assessment of direct relationships between metabolic and vascular risk factors and WMH burden. However, linear models do not account for complex interdependencies or potential mediating pathways that may underlie these associations—particularly the possibility that metabolic factors exert their influence on WMH indirectly via vascular changes such as atherosclerosis. Therefore, in the next stage of analysis, we specified structural equation models (SEMs), which enabled us to model both direct and indirect effects simultaneously, incorporate latent constructs, and formally test hypothesized pathways. The SEM framework provided a more nuanced understanding of the underlying mechanisms and allowed for the evaluation of the overall model fit to the observed data. We used the SEMopy package in Python (15). Additionally, information on asymptomatic carotid atherosclerosis was included in the model to account for subclinical vascular pathology. The model included several latent variables, each defined based on theoretically and clinically relevant observed indicators: glucose metabolism disorders (including prevalence of diabetes mellitus or fulfilling two definitions of prediabetes—IGT and IFG and glycated hemoglobin), hypertension–lipid composite (including hypertension and lipid profile), and carotid atherosclerosis composite (including carotid plaque presence and mean intima-media thickness in both sides). The model specifies that glucose metabolism disorders are predicted by an observed variable: visceral mass. In other words, visceral mass was hypothesized to have a direct effect on the latent variable. The relationships between latent variables and WMH were specified based on existing pathophysiological knowledge. Measurement models for each latent construct were evaluated, and path coefficients were estimated to determine the direct and indirect effects on WMH. The model controlled for key demographic covariates, such as age and sex. Model estimation was performed using maximum likelihood estimation (MLE) as implemented in SEMopy. To assess model fit, we considered the following fit indices:

- Comparative fit index (CFI) and goodness-of-fit index (GFI): we required values above 0.90 to indicate good fit.
- Root mean square error of approximation (RMSEA): we aimed for values below 0.08 to ensure an acceptable fit.

Statistical hypotheses were verified at a 0.05 significance level. The calculation was performed using STATA 16 and Python (version 3.10.9) with the following libraries: pandas version 2.2.3 (data manipulation), scipy version 1.9.3 (statistical testing), sklearn version 1.0.2 (predictive modeling), and SEMopy version 2.3.11 (SEM) (15).

## 3 Results

**Tables 2**–**5** present the characteristics of the study population. The results of the *post-hoc* test are presented in **Supplementary Materials** in **Supplementary Table 1**. Median age was 45 (36–58) years old; 341 (46.39%) individuals were men, 256 (34.92%) had hypertension, 383 (52.18%) had hypercholesterolemia, 58 (7.89%) had diabetes mellitus, 345 (46.94%) had prediabetes based on ADA guidelines, 163 (22.18%) had metabolic syndrome based on 2022 guidelines, median body mass index was 25.8 (22.9–29.3) kg/m^2^, and 148 (20.14%) individuals were obese. **Table 6** presents the association between clinical data and percentile of WMH with adjustment by age and gender.

**Table 2:** Descriptive statistics for population divided into 3 groups based on white matter hyperintensities burden quartiles—demographic and white matter hyperintensities data.

Variables	Analyzed population, *n* = 735	Groups based on white matter hyperintensity volume-to-white matter volume ratio quartiles			*P*
		Q1 + Q2, *n* = 367	Q3, *n* = 184	Q4, *n* = 184	
Age, years	45 (36–58)	38 (32–46)	48 (39–59)	61 (51–66)	<0.001 a^,b,c^
Gender, male	341 (46.39)	172 (46.87)	82 (44.57)	87 (47.28)	0.844
White matter hyperintensity volume, mm^3^	95.2 (2.1–482.0)	2.1 (0.2–29.2)	221.3 (148.5–325.6)	1,231.1 (701.3–2393.9)	

Data are presented as median and interquartile range for continuous variables, and number and percentage for categorical variables.

Comparison of variables between the subgroups was performed using the Dunn test for continuous variables and as pairwise comparisons between groups: Q1 + Q2 vs. Q3, Q1 + Q2 vs. Q4, and Q3 vs. Q4; the same letters in each row represent significant differences at *p* <⁠ 0.05 after the Bonferroni correction.

a Between Q1 + Q2 and Q3.

b Between Q1 + Q2 and Q4.

c Between Q3 and Q4.

Q, quartile.

The data presented in **Tables 3**-**5** are structured analogously to those shown in **Table 2**.

**Table 3:** Descriptive statistics for population divided into 3 groups based on white matter hyperintensity burden quartiles—glycemic and metabolic disturbance data.

Variables	Analyzed population, *n* = 735	Groups based on white matter hyperintensity volume-to-white matter volume ratio quartiles			*P*
		Q1 + Q2, *n* = 367	Q3, *n* = 184	Q4, *n* = 184	
Fasting glucose, mg/dL	99 (93–105)	96 (91–103)	99 (93–105)	104 (98–113)	<0.001 a^,b,c^
Glucose 120 min after oral glucose tolerance test, mg/dL (*n* = 684)	119 (101–138)	113 (98–131)	120 (101–142)	127 (111–155)	<0.001 a^,b,c^
Glycated hemoglobin, %	5.4 (5.1–5.6)	5.2 (5.0–5.5)	5.4 (5.2–5.7)	5.6 (5.3–5.8)	<0.001 a^,b,c^
Fasting C-peptide	2.29 (1.82–3.04)	2.16 (1.72–2.81)	2.4 (1.87–3.00)	2.48 (1.91–3.36)	<0.001 a^,b^
Diabetes mellitus (history of or newly diagnosed), *n* (%)	58 (7.89)	11 (3.00)	9 (4.89)	38 (20.65)	<0.001 ^b,c^
Prediabetes based on ADA guidelines	345 (46.94)	158 (43.05)	90 (48.91)	97 (52.72)	0.003 ^b^
Isolated impaired glucose tolerance based on ADA guidelines	46 (6.27)	27 (7.50)	11 (6.04)	8 (4.40)	0.37
Isolated impaired fasting glucose based on ADA guidelines	208 (28.34)	106 (28.96)	47 (25.54)	55 (29.89)	0.61
Prediabetes II group based on ADA guidelines	91 (12.38)	25 (6.83)	32 (17.39)	34 (18.48)	<0.001 a^,b^
Metabolic syndrome based on 2022 guidelines	163 (22.18)	50 (13.62)	47 (25.54)	66 (35.87)	<0.001 a^,b,c^

**Table 4:** Descriptive statistics for population divided into 3 groups based on white matter hyperintensity burden quartiles—cardiovascular data.

Variables	Analyzed population, *n* = 735	Groups based on white matter hyperintensity volume-to-white matter volume ratio quartiles			*P*
		Q1 + Q2, *n* = 367	Q3, *n* = 184	Q4, *n* = 184	
Total cholesterol, mg/dL	189 (116-218)	186 (164-211)	190 (170-218)	199 (174-228)	0.002 ^b^
Triglycerides, mg/dL	93 (68-132)	88 (65-126)	94 (68-136)	98 (75-135)	0.104
Hypertension (history of or newly diagnosed), *n* (%)	256 (34.92)	93 (25.48)	68 (36.96)	95 (51.63)	<0.001 a^,b,c^
Hypercholesterolemia (history of or newly diagnosed), *n* (%)	383 (52.18)	159 (43.44)	101 (54.89)	123 (66.85)	<0.001 a^,b^
Systolic blood pressure, mmHg	122 (110–134)	119 (107–130)	123 (111–135)	128 (116–138)	<0.001 a^,b,c^
Diastolic blood pressure, mmHg	81 (74–88)	80 (73–87)	81 (76–89)	82 (75–90)	0.014 ^a,b^
Systolic blood pressure > 140 mmHg or diastolic blood pressure > 90 mmHg, *n* (%)	177 (24.10)	67 (18.26)	50 (27.17)	60 (32.79)	<0.001 a^,b^
Carotid plaques, *n* (%)	292 (39.73)	91 (24.80)	69 (37.50)	132 (71.74)	<0.001 a^,b,c^
Mean intima-media thickness, mm	0.60 (0.53–0.69)	0.56 (0.52–0.62)	0.62 (0.55–0.70)	0.68 (0.61–0.80)	<0.001 a^,b,c^

**Table 5:** Descriptive statistics for population divided into 3 groups based on white matter hyperintensity burden quartiles—body composition data.

Variables	Analyzed population, *n* = 735	Groups based on white matter hyperintensity volume-to-white matter volume ratio quartiles			*P*
		Q1 + Q2, *n* = 367	Q3, *n* = 184	Q4, *n* = 184	
Height, cm	170 (164–178)	172 (166–180)	170 (163–177)	167 (161–175)	<0.001 a^,b,c^
Weight, kg	76 (65–89)	75 (64–89)	77 (65–86)	77 (68–88)	0.728
Body mass index, kg/m^2^	25.8 (22.9–29.3)	25.4 (22.6–28.2)	25.8 (22.8–29.5)	27.7 (24.1–30.9)	<0.001 ^b,c^
Body mass index ≥ 30.00, *n* (%)	148 (20.14)	50 (13.62)	43 (23.37)	55 (29.89)	<0.001 a^,b^
Waist-to-hip ratio	0.86 (0.79–0.94)	0.85 (0.77–0.92)	0.86 (0.81–0.94)	0.90 (0.83–0.97)	<0.001 a^,b,c^
Waist-to-hip ratio ≥ 0.85 cm for women or ≥ 0.90 for men, *n* (%)	327 (44.49)	135 (36.78)	82 (44.57)	110 (59.78)	<0.001 a^,b,c^
Waist circumference, cm	86 (77–96)	83 (75–94)	85 (77–97)	90 (81–100)	<0.001 ^b,c^
Waist circumference > 88 cm for women or > 102 for men, *n* (%)	174 (23.67)	56 (15.26)	47 (25.54)	71 (38.59)	<0.001 a^,b,c^
Visceral mass, kg	0.93 (0.42–1.67)	0.73 (0.33–1.46)	0.94 (0.39–1.75)	1.32 (0.69–1.96)	<0.001 a^,b,c^

**Table 6:** Linear regression models: association between clinical variable and WMH after adjustment by age and gender.

Variables	Model—adjusted by age and gender		
	β	*P*	*R* ^2adj.^
Fasting glucose, mg/dL	0.055	0.07	0.3243
Glucose 120 min after oral glucose tolerance test, mg/dL (*n* = 684)	0.070	0.030	0.3120
Total cholesterol, mg/dL	−0.033	0.27	0.3229
Glycated hemoglobin, %	0.030	0.31	0.3227
Fasting C-peptide	−0.061	0.06	0.3246
Hypertension (history of or newly diagnosed), *n* (%)	0.045	0.17	0.323
Hypercholesterolemia (history of or newly diagnosed), *n* (%)	−0.031	0.35	0.3229
Diabetes mellitus (history of or newly diagnosed), *n* (%)	0.077	0.017	0.3274
Metabolic syndrome based on 2022 guidelines	0.019	0.57	0.3223
Systolic blood pressure, mmHg	0.063	0.07	0.3242
Diastolic blood pressure, mmHg	0.030	0.31	0.3222
Systolic blood pressure > 140 mmHg or diastolic blood pressure > 90 mmHg, *n* (%)	0.0548	0.07	0.3241
Carotid plaques, *n* (%)	0.052	0.22	0.3237
Mean intima-media thickness, mm	0.100	0.028	0.3267
Height, cm	−0.081	0.08	0.3267
Body mass index, kg/m^2^	−0.056	0.10	0.3247
Body mass index ≥ 30.00, *n* (%)	0.001	0.97	0.3220
Waist-to-hip ratio	0.011	0.82	0.3221
Waist-to-hip ratio ≥ 0.85 cm for women or ≥ 0.90 for men, *n* (%)	−0.001	0.99	0.3220
Waist circumference, cm	−0.071	0.07	0.3251
Waist circumference > 88 cm for women or > 102 for men, *n* (%)	−0.002	0.96	0.3049
Visceral mass, kg	−0.048	0.226	0.3268

The study population was stratified into three groups based on quartiles of WMH’s burden. Groups Q1 and Q2 were combined and compared to Q3 and Q4. Participants in the highest quartile were significantly older compared to those in Q3 and Q1–Q2 [median age: 61 (51–66) vs. 48 (39–59) and 38 (32–46) years, respectively; *p* < 0.001]. No significant differences in sex distribution were observed across the groups (*p* = 0.844).

Fasting glucose, 2-h OGTT glucose, and HbA1c rose progressively with WMH burden (all *p* < 0.001). Fasting glucose was highest in Q4 [104 (98–113) mg/dL] vs. Q3 [99 (93–105)] and Q1–Q2 [96 (91–103)], with Q3 > Q1–Q2. Similar trends were seen for 2-h OGTT [Q4: 127 (111–155) vs. Q3: 120 (101–142) vs. Q1–Q2: 113 (98–131) mg/dL] and HbA1c [5.6% (5.3%–5.8%) vs. 5.4% (5.2%–5.7%) vs. 5.2% (5.0%–5.5%)]; all comparisons were significant. Fasting C-peptide was higher in Q4 [2.48 (1.91–3.36) ng/mL] than in Q1–Q2 [2.16 (1.72–2.81); *p* < 0.001] and in Q3 [2.27 (1.75–3.00); *p* = 0.04] vs. Q1–Q2, with no Q3–Q4 difference. Diabetes prevalence rose with WMH burden, reaching 20.7% in Q4 vs. 4.9% in Q3 and 3.0% in Q1–Q2 (*p* < 0.001), with Q4 differing significantly from both Q3 and Q1–Q2. Prediabetes I + prediabetes II prevalence did not differ significantly. Prediabetes II was more frequent in Q4 (18.5%) and Q3 (17.4%) than in Q1–Q2 (6.8%; both *p* < 0.001), while isolated IGT, isolated IFG, and either condition alone showed no significant differences. After adjustment for age and gender, only 2-h OGTT glucose (β = 0.070, *p* = 0.03, *R*^2^ = 0.312) and history of diabetes or diagnosis of diabetes mellitus (β = 0.077, *p* = 0.02, *R*^2^ = 0.327) retained significant association with percentile of WMH. The connection between fasting glucose level and percentile of WMH was attenuated and did not approach statistical significance (β = 0.055, *p* = 0.07, *R*^2^ = 0.324) after considering age and gender influence.

Total cholesterol was higher in Q4 [199 (174–228) mg/dL] than in Q1–Q2 [186 (164–211); *p* = 0.002], with no Q3 differences. Hypercholesterolemia was more common in Q4 (66.9%) vs. Q3 (54.9%) and Q1–Q2 (43.4%; *p* < 0.001 and *p* = 0.03, respectively). Triglycerides did not differ between analyzed groups (*p* = 0.104). The contribution of hypercholesterolemia was attenuated and no longer statistically significant after adjustment by age and gender.

Systolic blood pressure rose across quartiles, highest in Q4 [128 (116–138) mmHg] vs. Q3 [123 (111–135)] and Q1–Q2 [119 (107–130); *p* < 0.001]; diastolic blood pressure was modestly higher in Q4 [82 (75–90) mmHg] than in Q1–Q2 [80 (73–87)] and Q3 [81 (76–89); *p* = 0.01]. Elevated blood pressure prevalence increased from 18.3% (Q1–Q2) to 27.2% (Q3) and 32.8% (Q4; *p* < 0.001). Hypertension prevalence was highest in Q4 (51.6%) vs. Q3 (37.0%) and Q1–Q2 (25.5%; *p* < 0.001). The effect of high blood pressure was attenuated and no longer statistically significant after adjustment. Carotid ultrasound findings were strongly associated with WMH, as presented in our previous study (9).

Body mass index was greatest in Q4 [27.7 (24.1–30.9) kg/m²] vs. Q1–Q2 [25.4 (22.6–28.2)] and Q3 [25.8 (22.8–29.5); *p* < 0.001]; obesity prevalence was 29.9% in Q4 vs. 13.6% and 23.4% (*p* < 0.001). WHR was higher in Q4 [0.90 (0.83–0.97)] vs. Q1–Q2 [0.85 (0.77–0.92)] and Q3 [0.86 (0.81–0.94); *p* < 0.001], with central obesity more frequent (59.8% vs. 36.8% and 44.6%; *p* < 0.001). Waist circumference was greater in Q4 [90 (81–100) cm] vs. Q1–Q2 [83 (75–94)] and Q3 [85 (77–97); *p* < 0.001]; increased waist reached 38.6% in Q4 vs. 15.3% and 25.5% (*p* < 0.001). Visceral fat was highest in Q4 [1.32 (0.69–1.96) kg] vs. Q1–Q2 [0.73 (0.33–1.46)] and Q3 [0.94 (0.39–1.75); *p* < 0.001]. However, these associations became nonsignificant after adjustment for age and sex.

**Figure 2:**
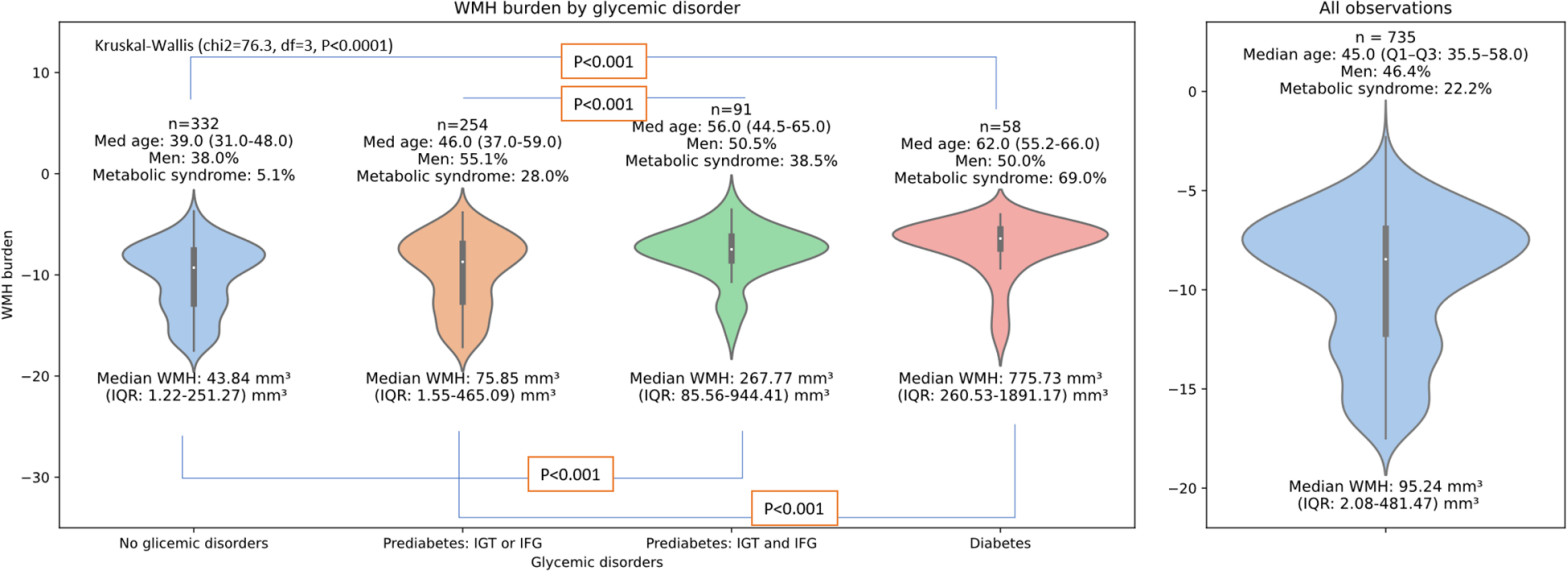
Violin plot illustrating the distribution of white matter hyperintensity (WMH) volume across glycemic groups. The plotted values are based on log-transformed WMH/WH ratio volume to improve visualization; however, statistical analysis and interpretation refer to the original data—WMH volume. A Kruskal–Wallis test revealed significant group differences (*p* < 0.001), with *post-hoc* Dunn’s test indicating pairwise significance between selected groups (*p* < 0.05).

The number of individuals with glycemic disorders was *n* = 403 (54.8%). **Figure 2** presented a violin plot depicting the burden of WMH across four glycemic disorder categories. Kruskal–Wallis test analysis revealed significant differences in WMH burden (χ^2^ = 76.3, df = 3, *p* < 0.001), percentiles of WMH (χ^2^ = 76.4, df = 3, *p* < 0.001), and WMH volume (χ^2^ = 74.8, df = 3, *p* < 0.001) between the groups. Further *post-hoc* analysis, using Dunn’s test with Bonferroni correction, indicated the following for WMH volume, WMH burden, and percentile of WMH:

- The normoglycemia group differed significantly from both the prediabetes II group (*p* < 0.001) and the diabetes mellitus group (*p* < 0.001).
- The prediabetes I group differed significantly from both prediabetes II group (*p* < 0.001) and the diabetes mellitus group (*p* < 0.001).

Additionally, as glucose disturbances increased, so did the median age (χ^2^ = 154.1, df = 3, *p* < 0.001), the proportion of men (χ^2^ = 33.6, df = 4, *p* < 0.001), and the proportion of individuals with metabolic syndrome (χ^2^ = 149.6, df = 4, *p* < 0.001). Next, we defined three linear models and presented them in **Table 7**. Each of Models 1–3 included a different binary glycemic status variable as exposure in the same overall population: Model 1 considered diagnosed diabetes mellitus, Model 2 included diabetes or prediabetes II, and Model 3 incorporated diabetes or any prediabetes definition (prediabetes I or prediabetes II). Across all models, age (*p* < 0.001) was the strongest and most significant predictor of WMH percentiles. Model 1 showed that diagnosed diabetes mellitus was independently associated with significantly higher WMH percentiles (β = 0.074, *p* = 0.022), adjusting for age, sex, and hypertension. Model 2 extended this finding by demonstrating that individuals with either diabetes mellitus or prediabetes II had significantly higher WMH percentiles (β = 0.092, *p* = 0.006), after adjusting for the same covariates. In Model 3, which included individuals with any form of glycemic abnormality (diabetes or prediabetes defined as IGT or IFG or both), the association with WMH percentiles was no longer statistically significant (β = −0.025, *p* = 0.469).

**Table 7:** Linear regression models: Models 1–3: assessment of the relationship between glucose disturbances groups and WMH percentiles, adjusting for age, sex, and hypertension.

	*b*	SE		*t*	*P*
Model 1: *F*(4,729) = 88.91, *p* < 0.001, *R*^2adj^. = 0.324					
Age, years	1.111	0.070	0.535	15.98	<0.001
Male, yes or no	1.189	1.817	0.021	0.65	0.513
Diabetes mellitus, yes or no	7.855	3.425	0.074	2.29	0.022
Hypertension, yes or no	2.091	2.021	0.035	1.03	0.301
Dependent variable: percentile of white matter hyperintensities					
Model 2: *F*(4,729) = 89.80, *p* < 0.001, *R*^2adj^. = 0.326					
Age, years	1.085	0.071	0.522	15.21	<0.001
Male, yes or no	1.065	1.815	0.018	0.59	0.558
Diabetes mellitus or prediabetes II, yes or no	6.628	2.393	0.092	2.77	0.006
Hypertension, yes or no	1.795	2.026	0.030	0.89	0.376
Dependent variable: percentile of white matter hyperintensities					
Model 3: *F*(4,729) = 87.16, *p* < 0.001, *R*^2adj^. = 0.312					
Age, years	1.166	0.072	0.562	16.25	<0.001
Male, yes or no	1.442	1.844	0.025	0.78	0.434
Diabetes mellitus or prediabetes I or prediabetes II, yes or no	−1.452	2.003	−0.025	−0.72	0.469
Hypertension, yes or no	3.025	2.059	0.050	1.47	0.142

**Figure 3:**
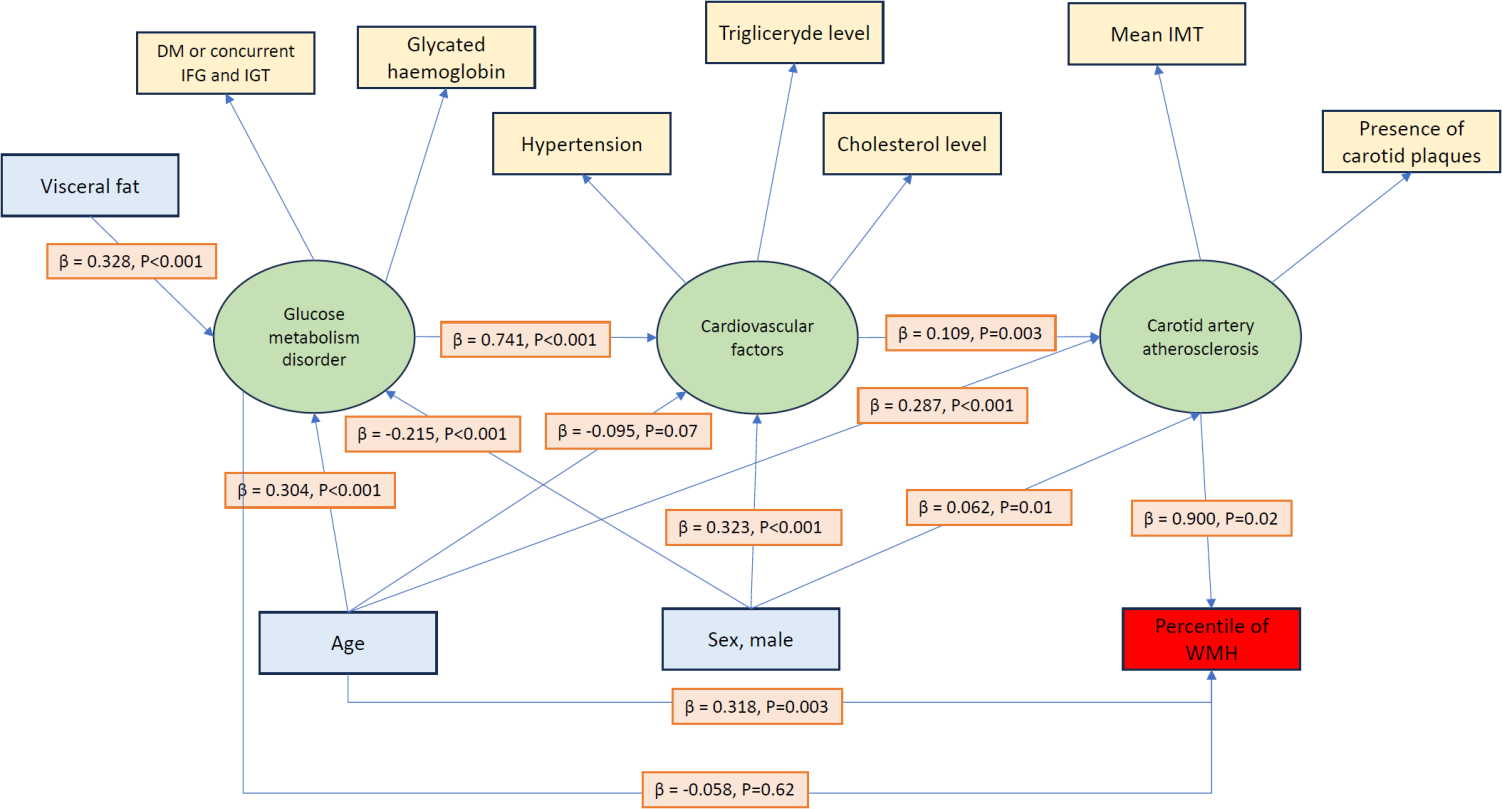
Final structural equation model (SEM) showing relationships between glucose metabolism disorders, cardiovascular factors, carotid atherosclerosis, and WMH burden. Latent variables are in green, observed variables are in yellow, covariates are in blue, and outcomes are in red. Standardized regression coefficients (β) with *p*-values are indicated on arrows. Model fit: CFI = 0.937, GFI = 0.923, RMSEA = 0.074.

**Figure 3** presents the final SEM, which includes three latent variables:

- Glucose metabolism disorder composite (incorporating diagnosed diabetes status, simultaneous fulfillment of both IGT and IFG criteria, and glycated hemoglobin levels),
- Hypertension–lipid composite (comprising hypertension, triglycerides, and total cholesterol levels), and
- Carotid artery atherosclerosis composite (measured by carotid intima-media thickness and the presence of carotid plaques).

**Table 8** presents factor loadings of latent construct. The model also accounts for potential moderating effects of age and sex on these relationships and their impact on percentile of WMH. Model fit indicators: CFI = 0.937, GFI = 0.923, RMSEA = 0.074.

**Table 8:** Measurement model details for SEM: latent variables, observed indicators, and factor loadings.

Latent variable	Observed variable	Factor loading (β)	*P*-value
Glucose metabolism disorders	DM or concurrent IFG and IGT	0.377	<0.01
Glucose metabolism disorders	Glycated hemoglobin	1	–
Cardiovascular factors	Hypertension	0.569	<0.01
Cardiovascular factors	Triglyceride level	1	–
Cardiovascular factors	Cholesterol level	0.719	<0.01
Carotid artery atherosclerosis	Mean IMT	2.329	<0.01
Carotid artery atherosclerosis	Plaques (presence)	1	–

Age showed significant positive associations with atherosclerosis (β = 0.29, *p* < 0.001), glucose metabolism disorders (β = 0.30, *p* < 0.001), and percentiles of WMH (β = 0.32, *p* = 0.003). Glucose metabolism disorders had an effect on hypertension–lipid composite (β = 0.74, *p* < 0.001), which in turn influenced carotid artery atherosclerosis (β = 0.11, *p* = 0.003). Importantly, atherosclerosis significantly associated with percentile of WMH (β = 1.02, *p* = 0.04). Additionally, visceral fat mass was associated with glucose metabolism disorders (β = 0.33, *p* < 0.001).

Sex also played a significant role: men showed higher frequency of CVR factors (β = 0.32, *p* < 0.001) and atherosclerosis (β = 0.06, *p* = 0.01). Age and sex thus emerged as important factors within the model.

Metabolic syndrome (based on the 2022 guidelines) was present in 35.9% of individuals in Q4, compared to 25.5% in Q3 and only 13.6% in Q1–Q2 (*p* < 0.001). All between-group comparisons were statistically significant, indicating a robust association between increasing metabolic syndrome and WMH burden.

A total of 163 individuals with metabolic syndrome were identified (22.2%). Of those, *n* = 146 (89.57%) also had glucose metabolism disturbances. A significant difference in WMH burden was observed between the group without metabolic syndrome and the group with metabolic syndrome by using the Mann–Whitney test (*z* = −5.7, *p* < 0.001). Furthermore, individuals with metabolic syndrome were older than those without (*z* = −9.1, *p* < 0.001); however, the proportion of men did not differ significantly (χ^2^ = 0.61, *p* = 0.44). In the linear regression analysis (Model 4 from **Table 9**), metabolic syndrome was included as a binary predictor of WMH percentiles, alongside age and sex. While age remained a strong and significant predictor (β = 0.563, *p* < 0.001), metabolic syndrome did not show a statistically significant association with WMH burden when accompanied by age in the model (β = 0.019, *p* = 0.566). Similarly, sex was not significantly related to WMH in this model. These results suggest that, despite the higher WMH burden observed in individuals with metabolic syndrome in univariate comparisons, metabolic syndrome itself may not independently predict WMH severity when adjusting for age and sex.

**Table 9:** Linear regression models: Model 4: assessment of the relationship between metabolic syndrome and WMH percentiles, adjusting for age and sex.

	*b*	SE		*t*	*P*
Model 4: *F*(3,731) = 115.90, *p* < 0.001, *R*^2adj^. = 0.320					
Age, years	1.171	0.068	0.563	17.32	<0.001
Male, yes or no	1.704	1.772	0.029	0.96	0.336
Metabolic syndrome, yes or no	1.293	2.251	0.019	0.57	0.566

Dependent variable: percentile of white matter hyperintensities.

## 4 Discussion

Our study demonstrates that even apparently healthy individuals fulfilling the criteria of both IGT and IFG, which are intermediate states between normoglycemia and diabetes, have elevated WMHs and, hence, present a higher risk of dementia in the future. Lack of independence from age and gender association between isolated prediabetic states does not exclude its effects, but suggests possible requirement for more time or more severe glycemic disturbances. After comprehensive modeling including carotid atherosclerosis, the association becomes indirect; nevertheless, the findings indicate that prediabetes diagnosed during OGTT may facilitate the identification of patients at risk of developing WMH. Such alterations should be regarded as early warning markers, warranting the implementation of preventive strategies aimed not only at mitigating the long-term risk of diabetes mellitus but also at decelerating age-related cerebral decline.

Univariate analysis presents the association between fat distribution parameters, obesity, and WMH presence. Simultaneously, our multivariate analysis findings revealed a significant positive association between aging, impaired glucose metabolism, metabolic disturbances, hypertension, carotid atherosclerosis, and the volume of WMH. These results are consistent with the hypothesis that metabolic and vascular factors play a crucial role in the development of cerebral small vessel disease and are consistent with the results from other studies (3, 4, 16, 17).

Importantly, our findings suggest that with age, glucose disturbances are accompanied by an increasing prevalence of metabolic syndrome. Additionally, our results present that glucose disturbances are associated with worse blood pressure control and lipid profile. Uncontrolled hypertension and glucose disturbances impair the elasticity of large arteries, which increases mechanical stress on the microvasculature of the brain, making small vessels more susceptible to injury (18). Finally, damaged arteries promote microvascular damage such as WMHs in the brain. We cannot clearly state how aging affects development of WMH—whether it is the process of arterial degeneration or the accumulation of CVR factors, or, most likely, both.

The analysis of WMH burden across glycemic disorder groups demonstrates a graded association, with the highest WMH volume observed in individuals with diabetes, followed by prediabetes group II, then prediabetes group I, and the lowest burden in participants with normoglycemia. Notably, when prediabetes was defined based on a single criterion (prediabetes group I), no statistically significant association with WMH burden was detected. This finding may indicate that early or mild impairments in glucose metabolism are insufficient to produce detectable WM changes, possibly due to a shorter duration or lesser extension of exposure to metabolic disturbances. Furthermore, the prediabetes I group tended to be younger, which might have contributed to lower cumulative exposure to both metabolic and aging-related mechanisms underlying WMH development. Grosu et al. (19) reported that the effect of glycemic disorders on WMH appears to be influenced more by post-load glucose levels. In our population, 137 (18.64%) individuals had IGT and 91 (66.42%) of them also IFG. The meta-analysis prepared by Liu et al. (20) suggests that, within the population with prediabetes, individuals fulfilling the definition of both IGT and IFG criteria exhibit the highest risk of developing diabetes in the future. Diabete mellitus is an known factor associated with the progression of WMH, but our study extends this association to the population not yet diagnosed with diabetes, but with prediabetes and a high risk of developing one (fulfilling two independent criteria of prediabetes). Garfield et al. reported that prediabetes and undiagnosed or known diabetes conferred higher WMH volumes in comparison to individuals with normoglycemia. However, their study group contains individuals with Alzheimer’s dementia and vascular dementia (16), whose presence may significantly influence the observed phenomenon and cannot be compared to a population of apparently healthy individuals.

Structural equation modeling provided further insight into these interrelationships. Specifically, visceral adipose tissue emerges as a key driver of impaired glucose metabolism. In turn, deterioration in glucose metabolism significantly contributes to a poorer cardiovascular profile, marked by elevated blood pressure and dyslipidemia. Lampe et al. (21) reported that WMH burden is increased in individuals with obesity with high visceral fat accumulation. Veldsman et al. (22) indicate WHR as a significant risk factor associated with WMH. We do not observe a direct relationship between fat accumulation and WMH volume; however, our findings suggest a role of abdominal fat. The younger study population might be the reason why our results are different from the above studies; the median age in the Lampe study is 64.7 years, whereas it is 63.5 years in the Veldsman study and 45 years in our study. Additionally, neither Veldsman nor Lampe excluded severe cardiovascular diseases, similar to our study.

The cross-sectional and longitudinal study prepared by Wang et al. based on the UK Biobank cohort assessed the exposition of central obesity to WMH volume (23). Their results suggest that both current and past obesity are significantly associated with increased WMH volume. In our study, it was difficult to assess the exposition of obesity, because our other study presented that 21.73% of individuals were unaware of their overweight or obesity status (24). Analysis was difficult, because it was based on subjective self-reported data.

Finally, these findings suggest that visceral adiposity, impaired glucose metabolism, and cardiometabolic factors (i.e., hypertension and dyslipidemia) indirectly promote the progression of WMH.

### 4.1 Limitations

Because of the cross-sectional character of the study, we were unable to assess how long newly diagnosed diabetes, prediabetes, or obesity has been affecting the brain. Therefore, it is important to continue the prospective observation in the study.

The significant imbalance in numbers between participants with obesity and those without might limit the precision of estimates of comparative analysis. This might result in difficulties in detecting significant differences.

We assessed the global WMH volume, not on location in each brain structure, and we did not divide WMH as periventricular WMHs or deep WMHs.

MRI is an additional examination that is more often requested by younger and potentially healthier people (because of the exclusion criteria), which means that the proportion of individuals with diabetes mellitus is lower than the proportion in the Bialystok PLUS population (participants with MRI examination 8.89% vs. participants without MRI examinations 16.53%, *p* < 0.001). Also, this is a cross-sectional study; thus, we cannot make causal inference; we can only present associations.

### 4.2 Conclusions

The findings suggest a clear association between higher WMH burden and increased CVR factors, especially glucose metabolism disorders. These results underscore the importance of managing metabolic and CVR factors to potentially mitigate the risk of developing WMH and associated neurological complications.

Our analysis shows that diabetes mellitus and prediabetes are significantly associated with increased WMH. Moreover, SEM analysis revealed that visceral fat plays a pivotal role by contributing to glycemic abnormalities, which in turn mediate the effect on WMH burden.

Overall, our results support a model in which metabolic and vascular health are deeply interconnected and together modulate the risk of brain aging and small vessel disease. Preventive strategies targeting visceral obesity, glucose metabolism management, and cardiometabolic risk factors may thus be essential to mitigating WMH burden and preserving brain health later in life.

## Data Availability

The data analyzed in this study is subject to the following licenses/restrictions: The datasets are not publicly available because the individual privacy of the participants should be protected. Data are however available from the corresponding author on reasonable request. Requests to access these datasets should be directed to Bialystok PLUS; https://bialystok.plus/dokument/.
