# Supplementary material for: The impact of glucose and metabolic disturbances on white matter hyperintensity volume in apparently healthy adults
Source: Front Endocrinol (Lausanne). 2026 Mar 18;17:1789777. doi: 10.3389/fendo.2026.1789777 (PMC13038537; doi:10.3389/fendo.2026.1789777)
Supplement: Supplementary file 1 [file Table1.docx]

# Post-hoc analysis

Table S1. Results of post-hoc analysis with using Dunn’s test with Bonferroni correction for continuous variables or $\chi^{2}$ for category variables.

| Variables | Kruskall-Wallis or $\chi^{2}$, P | Q1+Q2 vs Q3, P | Q1+Q2 vs. Q4, P | Q3 vs Q4, P |
| --- | --- | --- | --- | --- |
| Age, year | <0.001 | <0.001 | <0.001 | <0.001 |
| Fasting glucose, mg/dL | <0.001 | 0.028 | <0.001 | <0.001 |
| Glucose 120 min after oral glucose tolerance test, mg/dL (n=684) | <0.001 | 0.011 | <0.001 | 0.0061 |
| Total cholesterol, mg/dL | 0.002 | 0.12 | <0.001 | 0.18 |
| Glycated hemoglobin, % | <0.001 | <0.001 | <0.001 | 0.001 |
| Fasting C-peptide | <0.001 | 0.041 | <0.001 | 0.31 |
| Hypertension (history of or newly diagnosed), n (%) | <0.001 | 0.015 | <0.001 | 0.012 |
| Hypercholesterolemia (history of or newly diagnosed), n (%) | <0.001 | 0.030 | <0.001 | 0.06 |
| Diabetes Mellitus (history of or newly diagnosed), n (%) | <0.001 | 0.78 | <0.001 | <0.001 |
| Prediabetes based on ADA guidelines | 0.003 | 0.19 | 0.032 | 0.47 |
| Impaired glucose tolerance and isolated impaired fasting glucose based on ADA guidelines | <0.001 | <0.001 | <0.001 | 0.79 |
| Metabolic syndrome based on 2022 guidelines | <0.001 | 0.001 | <0.001 | 0.032 |
| Systolic blood pressure, mmHg | <0.001 | 0.029 | <0.001 | 0.013 |
| Diastolic blood pressure, mmHg | 0.014 | 0.028 | 0.009 | 1.00 |
| Systolic blood pressure > 140 mmHg or diastolic blood pressure > 90 mmHg, n (%) | <0.001 | 0.048 | <0.001 | 0.723 |
| Carotid plaques, n (%) | <0.001 | 0.006 | <0.001 | <0.001 |
| Mean Intima-media thickness, mm | <0.001 | <0.001 | <0.001 | <0.001 |
| Height, cm | <0.001 | 0.006 | <0.001 | 0.036 |
| Body mass index, kg/m^2^ | <0.001 | 0.151 | <0.001 | 0.008 |
| Body mass index > 30.00, n (%) | <0.001 | 0.012 | <0.001 | 0.16 |
| Waist-to-hip ratio | <0.001 | 0.022 | <0.001 | 0.014 |
| Waist-to-hip ratio ≥ 0.85 cm for women or ≥ 0.90 for men, n (%) | <0.001 | 0.23 | <0.001 | 0.009 |
| Waist circumference, cm | <0.001 | 0.15 | <0.001 | 0.004 |
| Waist circumference > 88 cm for women or > 102 for men, n (%) | <0.001 | 0.009 | <0.001 | 0.021 |
| Visceral mass, kg | <0.001 | 0.024 | <0.001 | 0.001 |
